# Study on the Spatial Differentiation of Public Health Service Capabilities of European Union under the Background of the COVID-19 Crisis

**DOI:** 10.3390/healthcare8040358

**Published:** 2020-09-24

**Authors:** Xuhui Ding, Zhongyao Cai, Wei Zhu, Zhu Fu

**Affiliations:** 1 School of Finance and Economics, Institute of Industrial Economics, Jiangsu University, Zhenjiang 212013, Jiangsu, China; dingxh@ujs.edu.cn; 2 School of Business, Hohai University, Changzhou 213022, Jiangsu, China; caizy99@163.com; 3 School of Economics, Lanzhou University, Lanzhou 730000, Gansu, China; 4 School of Economics and Management, Jiangsu University of Science and Technology, Zhenjiang 212003, Jiangsu, China; fuzhu666@hhu.edu.cn

**Keywords:** public health service capabilities, European Union, spatial differentiation, COVID-19 crisis

## Abstract

Access to public health services is a cause that benefits the people and concerns the vital interests of the people. Everyone has access to basic health care services. The continuous improvement in people’s health is an important indicator of the improvement in people’s quality of life. This paper selects data from the European Union (EU) on aspects of public health expenditure, medical care resources, and government emergency coordination capacity from the period 2008 to 2017. Principal component analysis and factor analysis are used to measure their public health service capacity scores and conduct a comparative analysis. On this basis, the TOBIT model is adopted to explore the driving factors that lead to the spatial differentiation of public health service capabilities, and to combine it with the data of the COVID-19 epidemic as of 8 August 2020 from the official announcements of the World Health Organization and governments for further thinking. The results indicate that the public health service capacity of countries in the EU is showing a gradual increase. The capacity in Western Europe is, in turn, higher than that of Northern Europe, Southern Europe and Eastern Europe. In addition, the overall capacity in Western Europe is relatively high, but it is not balanced and stable, while Northern Europe has remained stable and balanced at a high level. Population density, degree of opening up, education level, economic development level, technological innovation level, and degree of aging have a positive effect on public health service capabilities. The level of urbanization has a negative effect on it. However, in countries with strong public health service capabilities, the epidemic of COVID-19 is more severe. The emergence of this paradox may be related to the detection capabilities of countries, the high probability of spreading thCOVID-19 epidemic, the inefficient implementation of government policy, the integrated system of the EU and the adverse selection of youth. This paper aims to improve the ability of the EU to respond to public health emergencies, improve the utilization of medical and health resources, and better protect people’s health from the perspective of public health service capacity.

## 1. Introduction

According to the official announcements of the World Health Organization and governments, the epidemic of COVID-19 is spreading rapidly in European Union (EU) countries. Before 8 August 2020, fifteen countries in the EU had a cumulative number of diagnoses of more than ten thousand, especially Spain, which ranked 10th in the world with a cumulative number of more than 350,000. The cumulative number of diagnoses in Italy, France, and Germany exceeded 200,000. The prevention and control of the epidemic is also a test of the public health service capabilities of countries in the EU. However, countries with high levels of medical care, such as Italy, have a fatality rate of 14.09%. The fatality rate in high-welfare and high-income countries such as Sweden also reached 7.00%. The cumulative diagnosis rate of COVID-19 in Luxembourg is higher than 1%. With the increasingly serious epidemic situation, people’s health is seriously threatened, the social economy has suffered huge losses, and the government’s prestige has also been questioned. On 30 January 2020, the director of the World Health Organization publicly announced that the epidemic of COVID-19 constituted a public health emergency of international concern. According to the treaty, public health services are mainly the jurisdiction of each country, so countries in the EU have adopted differentiated response measures to prevent and control the epidemic. However, some countries underestimated the severity of the COVID-19 epidemic. This public health crisis is an institutional crisis, exposing the problems of the public health service system and government intervention policies, exposing the ability to coordinate governance that is inconsistent with the integration of the EU, and exposing the mismatched relationship between public health service capacity and the economic strength of countries [1]. Those problems show that it is urgent for governments to take measures to coordinate and improve public health services from the perspective of the EU as a whole.

Experts at home and abroad have conducted various studies on public health services. Foreign experts pay more attention to evaluating public health services in the medical field for certain diseases, such as the evaluation of dental health services during the epidemic of COVID-19 [2]. Some experts discussed the public health crisis from the perspective of national epidemics [3]. Domestic experts mostly conduct research on the equalization of public health services. Wang Bo and Liu Jinsong et al. evaluated the public health service capabilities of some provinces in China, and conducted countermeasures for the equalization of public health services [4,5]. The development of public health services around the world is uneven. When evaluating public health service capabilities, existing research mostly selected indicators at the medical and health level and the allocation of medical resources, and used methods such as fuzzy evaluation method and cluster analysis, or selected relevant indicators for research. Guo Yuling used principal component analysis and cluster analysis to evaluate the medical and health level of provinces in China, from the perspective of medical and health resources, medical and health services and health security capabilities [6]. You Jianpeng adopted the synthetical index method to comprehensively analyze the medical service quality of county-level general hospitals in Guangxi Province [7]. Since the medical and health industry has the characteristics of multiple inputs and outputs, many studies used the DEA method to measure the efficiency of medical institutions and health services [8,9]. The above research has not considered the government level. The government’s emergency response is closely related to the coordination capacity and the regional public health service capacity, which is a crucial evaluation dimension [10].

There are few studies on how to quickly improve the capacity of public health services. For example, the WHO European Action Plan could strengthen public health services and capacities [11]. Taking Italy as an example, studies have shown that prevention programs can effectively strengthen Italy’s public medical capabilities and services [12]. The relevant research mostly considers issues from the aspects of medical staff and the medical system itself, but not from the macro-level economic development level, education level, and aging degree of the country. Hurjui, I. et al. pointed out the importance of medical staff in public health services [13]. Chu Ting et al. pointed out that strengthening the training of professional doctors’ service ability is an important way to improve the level of hospital health services [14]. In addition, the existing studies are aimed at individual countries or regions for analysis [15,16]. There is no horizontal comparison between countries. This paper first introduces the background of the EU’s public health services and a literature review of related studies. The second part introduces the construction principle of the model. In the third part, data are selected from the official announcements of the World Health Organization and governments on fifteen variables in the aspects of public health expenditure, medical care resources, and government emergency coordination capabilities in 27 countries of the EU from the period 2008 to 2017. Principal component analysis and factor analysis are used to measure the public health service capacity scores of EU countries and conduct comparative analysis. Then, this paper selects nine indicators of eight dimensions with Tobit model to explore the driving factors of public health service capacity. The empirical results are discussed in the fourth part. Fifth, based on the public health service capacity, comprehensively considering the current epidemic situation in each country, we try to explore the correlation between the two and present any further thinking, Finally, the conclusion and policy recommendations are given.

## 2. Model Construction

### 2.1. Evaluation Model

Public health services involve multiple subjects and a wide range of fields. This paper selects 15 variables from the dimensions of public health expenditure, medical care resources, and government emergency coordination capabilities to evaluate the public health service capabilities of countries in the EU. In order to extract key variable information, original multi-variable mass data information is reflected through fewer comprehensive variables [17]. Principal component analysis and factor analysis in the data dimensionality reduction method are adopted for evaluation. R in Equation (1) represents the correlation coefficient matrix. $K_{p}$ in Equation (2) represents the contribution rate of the principal component P. $F_{i}$ is the comprehensive score value. fip is the principal component value after normalization process [18].

(1) $$R={\left|\frac{1}{n-1}\sum_{t=1}^{m}X_{ti}*X_{ij}\right|}_{\left(m*m\right)}\;(i=1,2,\cdots ,m;j=1,2,\cdots ,m)$$

(2) $$K_{p}=\lambda_{p}/\sum_{p=1}^{k}\lambda_{p},\cdots F_{i}=\sum_{p=1}^{k}K_{p}f_{ip}$$

Factor analysis is used to find potential dominant factors. From the perspective of the effect of potential dominant factors on the whole, factor analysis is based on the correlation coefficient matrix ρ to extract the principal factors with the principal component method. Assume that the eigenvalue and eigenvector pair of the correlation coefficient matrix R is $\left({\hat{\lambda }}_{1},{\hat{\varepsilon }}_{1}),({\hat{\lambda }}_{2},{\hat{\varepsilon }}_{2}),\cdots ,({\hat{\lambda }}_{m},{\hat{\varepsilon }}_{m}\right)$, where the factor loading matrix is $\tilde{L}=\left[\sqrt{{\hat{\lambda }}_{1}{\hat{\varepsilon }}_{1}}\vdots \sqrt{{\hat{\lambda }}_{2}{\hat{\varepsilon }}_{2}}\vdots \cdots \vdots \sqrt{{\hat{\lambda }}_{P}{\hat{\varepsilon }}_{P}}\right]$, and the contribution rate of the i principal factor to the total variance is $w_{i}={\hat{\lambda }}_{i}/m$. Equation (3) is the basic model of factor analysis [19,20]. What’s more, assuming that the special factor ε is the error, the formula for calculating the factor score ${\hat{f}}_{i}$ and the comprehensive weighted total score $D_{j}$ of the j(j≤m) sample is shown in Equation (4).

(3) X−μ=LF+ε

(4) $${\hat{f}}_{i}={\left({\hat{L}}^{\prime }{\hat{L}}_{Z}\right)}^{-1}{{\hat{L}}^{\prime }}_{Z}Z_{i},\;D_{j}=\sum_{i=1}^{p}w_{i}f_{i}$$

### 2.2. Tobit Model

If the EU’s public health service capacity directly uses the super-efficiency value as the dependent variable to perform the least squares regression, the linear regression estimation under censorship includes additional computational complexity, and the parameter estimates will be biased and inconsistent. However, the Tobit model adopts the maximum likelihood method to effectively solve this problem [21]. This paper selects the Tobit model to test the driving factors of the EU’s public health service capacity. The model can solve the problem of model construction of restricted dependent variables or truncated dependent variables. The Tobit model has gradually been applied to the estimation of driving factors of efficiency indicators such as innovation efficiency and water efficiency, as shown in Equation (5). Among them, $y_{it}^{*}$ is the EU public health service capacity score, and each driving factor $x_{i}$ including core explanatory variables and control variables is introduced. $u_{i}$ is the individual effect in the panel model estimation. In addition, the Tobit panel model can further distinguish between random effects models and fixed effects models, depending on whether it is related to explanatory variables [22,23].

(5) $$y_{it}^{*}=x_{it}^{\prime }\beta +u_{i}+\varepsilon_{it}{\;S}_{t}\cdot y_{it}=\left\{ \begin{array}{ll} 0, & if\;y_{it}^{*}\leq 0\;\\ y_{it}^{*}, & if\;y_{it}^{*}>0 \end{array}\right.$$

## 3. Empirical Estimation

### 3.1. Evaluation of the EU Public Health Service Capacity

According to the existing research, public health service capacity is closely related to capital investment, resource allocation and government emergency coordination [24,25]. Appropriate investment and expenditure of public health funds and a good allocation of public health resources can maintain the stable development of the public health service industry. The government has an absolute leading role in the public health service industry. The government’s appropriate management and emergency coordination capabilities can promote the long-term development of the national public health service industry [26]. Therefore, public health service capacity is affected by many factors, such as public health funds, public health resources, and government emergency coordination capabilities. In order to better measure and evaluate the public health service capacity of countries in the EU, under the premise of following the principles of objectivity, systematization and operability, the following evaluation index system is constructed, as shown in Table 1. This paper selects 15 evaluation factors from three dimensions of public health expenditure, medical care resources, and government emergency coordination capabilities.

Based on the relevant data of 27 countries of the EU from 2008 to 2017, the comprehensive score table of public health service capacity was obtained by principal component analysis and factor analysis, as shown in Table 2 and Table A1 (Appendix A Table A1). There are significant differences in the scores of public health service capacity of countries in the EU from 2008 to 2017, but the overall trend is increasing year by year. It can be seen that the countries have gradually paid more attention to public health services in recent years [27]. The scores of Germany are generally higher than those of other countries, and the public health service capacity of relevant countries in Southern Europe and Eastern Europe needs to be further improved. The scores of principal component analysis and factor analysis are consistent with the reality of the countries. From the perspective of the results, the scores obtained by principal component analysis are more in line with the development trend of public health service capacity in countries in the EU. This paper uses the result of principal component analysis as the result of public health service capacity measurement.

### 3.2. Driving Factors

Public health service capacity is closely associated with the level of economic development, technological innovation, education, degree of opening up, and population density. In order to explore the driving factors of the public health service capacity of countries in the EU, the specific indicators selected are shown in Table 3 below. Population is the most significant feature of the size of a city. Population density directly affects the quality and demand of public health services, which is an important driving factor [28]. The globalization index includes the degree of openness to trade, capital flow, exchange of technology, labor mobility and cultural integration, which can fully represent the country’s degree of opening up. The education level selects the average years of education per capita as the explanatory variable. The popularity of education affects the development of talents and the entire region. Talents in the medical and health field are the core element. The economic development level selects indicators, including gross regional product (GDP) and per capita GDP. GDP is an intuitive manifestation of economic development, which directly affects the development of a country’s public health services. The level of urbanization reflects the proportion of urban population in the total population and the distribution. The level of technological innovation uses R&D investment as a percentage of GDP to indicate that social research and experimental development promote technological innovation, which is conducive to improving the national public health service capacity. The Gini coefficient is a common index to measure the gap between the rich and the poor, which means the uneven wealth of the country and the imbalance in residents’ income [29]. Public health services are mainly provided to the elderly. The proportion of elderly in the total population is an intuitive manifestation of the degree of aging. The gap between the rich and the poor and the degree of aging affect residents’ demand for public health services.

Combining the random effects and fixed effects theories of the Tobit model, this paper uses STATA to perform regression analysis. The regression results of factors affecting the public health service capacity of countries in the EU are shown in Table 4 below. The random effects and fixed effects results of the Tobit model meet the experimental standards. For the Tobit model analysis results, Hausman test was used, and the *p* value was significant and had fixed effect results. Therefore, the results of fixed effects are used as the criterion for judging factors affecting the EU’s public health service capacity. Of the nine variables in the analysis of fixed effects and random effects, five of them are significant in random effects, and seven of them are fixed effects, so they are more realistic.

## 4. Results Discussion

### 4.1. EU Public Health Service Capacity

From the perspective of the dynamic evolution of the public health service capacity of the 27 countries of the EU, there has been a gradual increase trend. This is closely related to the development of science and technology, people’s growing medical and health needs. The scores of some countries greatly improved between 2008 and 2017, especially Latvia, which is basically ranked in the bottom three, but its score increased by 36.79% from 38.19 in 2008 to 52.24 in 2017. This is because the total land area of Latvia is 62,046 square kilometers and the total population remains at about 2 million. The demand for public health service capacity is small, so it has a low base starting point and large room for improvement. However, there are significant differences in the capacity of public health services among different countries of the EU. As a country with a relatively high level of medical and health care, Germany’s public health service capacity score increased by 15.27% from 2008 to 2017 [30]. In particular, the score in 2008 was 82.69, which is about 1.5 times that of other countries. In terms of phases, the growth rate of countries from 2008 to 2010 was slower. The growth rate was relatively fast from 2011 to 2013, and it remained basically stable afterwards. It can be seen that countries in the EU have paid more and more attention to public health services.

**Figure 1:**
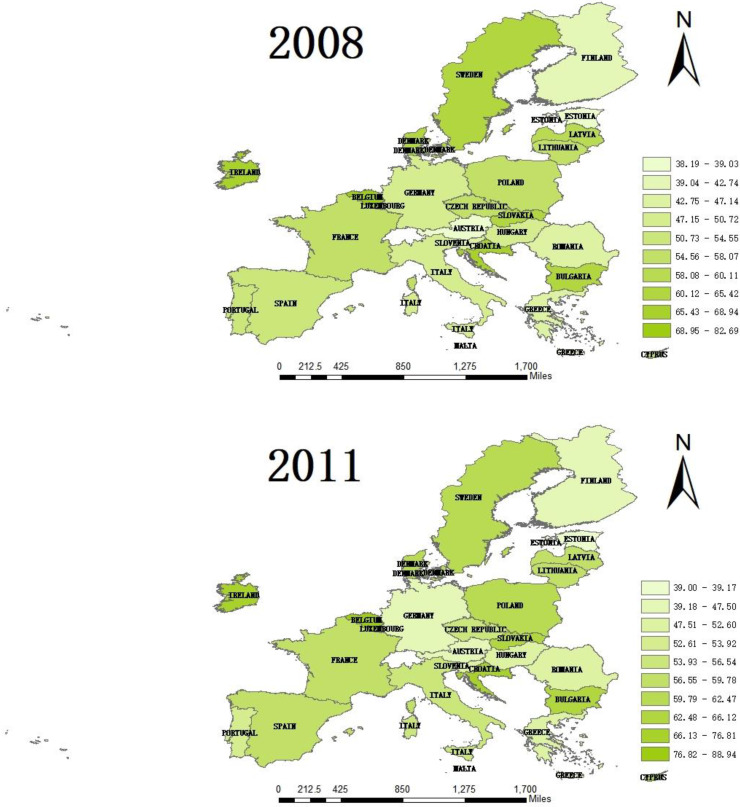

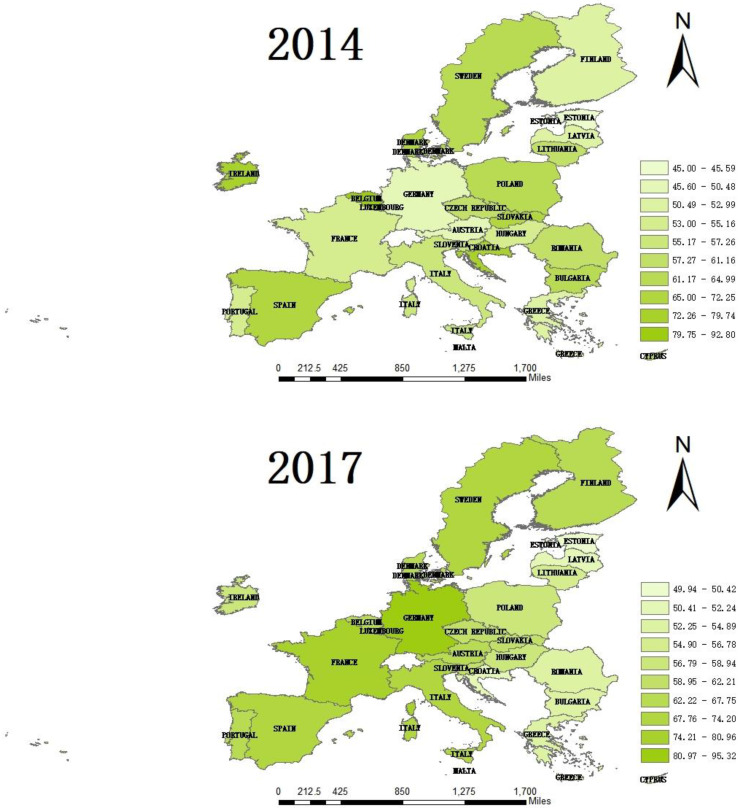
The color figures of public health service capacity scores of the EU in 2008, 2011, 2014 and 2017.

From the perspective of horizontal spatial differentiation, the absolute range of the public health service capacity of countries in the EU in 2008 and 2017 remained within 17, which was not greatly improved or decreased. Figure 1 shows that the countries with the highest public health capacity scores are Germany, France, Italy, the Netherlands, and Spain. Countries with weaker public health services are Cyprus, Estonia, Latvia and Bulgaria. The capacity of public health service has agglomeration characteristics in regional distribution. From the point of view of the average score of public health service capacity, the Western European countries (0.689) are sequentially higher than Northern Europe (0.658), Southern European (0.563) and Eastern European (0.539). This is closely related to the larger areas, larger populations and better economies of Western European countries. From the perspective of the standard deviation, Western Europe is the highest, followed by Southern Europe, Eastern Europe and Northern Europe. It can be seen that although the overall public health service capacity in Western Europe is relatively high, the development is not balanced and stable, while Northern Europe has remained stable and balanced at a high level.

### 4.2. Analysis of Driving Factors

From the above results, it can be seen that the Tobit model’s fixed effect regression results of population density, degree of opening up, education level, economic development level, urbanization level, technological innovation level and degree of aging are significant. They are driving factors for the public health service capacity of countries in the EU [31]. However, per capita GDP and the Gini coefficient did not pass the significance test, indicating that there is no direct correlation between per capita GDP and Gini coefficient and public health service capacity. Per capita GDP is the ratio of GDP to the country’s permanent population. It is inseparable from the country’s permanent population and cannot directly reflect the relationship between the economic development level and public health service capacity. The Gini coefficient is an important indicator to measure the income gap of residents. The medical and health resources of economically developed countries are mostly provided to people with a certain economic foundation. Low-income people cannot universally and comprehensively enjoy public health services [31]. This shows that the gap between the rich and the poor is not related to the capacity of public health services.

The increase in population density promotes the improvement in public health service capacity. Countries with high population density can trigger the agglomeration effect of funds, goods and talents. It strengthens the construction of public health services from the dimensions of funds, goods, and talents, thereby promoting the country’s public health service capabilities. On the one hand, countries with higher population densities have a stronger ability to serve the hinterland and can meet more public health service needs [32]. On the other hand, for areas with low population density and sparse population distribution, in order to ensure that people can enjoy basic public health services, the government will open small health service stations in relevant areas. While increasing the popularity of public health services, it has also dispersed the supply of public health materials, reduced the utilization rate of medical resources, and reduced the public health service capacity of relevant areas. However, in terms of population density, bigger is not better. Only by scientifically controlling the population density can we ensure the long-term development of national public health services.

Opening up plays a positive role in promoting public health service capabilities. The globalization index is an important index to measure the degree of opening up. It includes trade openness, capital flow, exchange of technology and ideas, labor mobility, and cultural integration. It not only promotes the accumulation of capital, technology, culture and labor, but also promotes the development of trade, affects the optimal allocation of production factors and resources, and promotes the improvement of public health service capabilities [33,34]. Among the 27 countries of the EU, France, Italy, Belgium and other countries have higher globalization indexes. France, which pursues a free trade policy, has a relatively high degree of opening up. Its medical and health system is an internationally recognized excellent system, covering medical, social services, and technological innovation. It has high operation efficiency and strong public health service capabilities. Therefore, countries in the EU need to continue to improve the level of opening up, promote economic cooperation, attract foreign investment, and introduce medical and health personnel, advanced technology and management experience, so as to enhance the public health service capacity [35].

The level of education can effectively promote the improvement in public health service capacity. From 2008 to 2017 in countries of the EU, the number of years of education basically showed a steady increase trend, which matched the gradual increase in public health service capacity. The higher the average number of years of education per capita, the higher the overall quality of the talents. They need a higher quality of life and are able to undertake public health services of a certain quality. To some extent, this has increased the demand for public health services and promoted the development of the public health service system. Therefore, we should continue to strengthen talent education and increase investment in education. It is necessary to focus on cultivating high-quality talents and promote the exchange and cooperation of relevant talents among the countries, so as to improve the public health service capabilities [36].

The total GDP is an important feature of national economic development level. The two influence and promote each other. The results show that the level of economic development of countries in the EU is positively correlated with the public health services’ capacities. The capacity of public health service is considered from the whole country. A country with stronger economic power can establish a more complete public health service system and have a higher public health service capacity [37,38]. In the EU, Germany, France, Italy, and Spain have higher levels of economic development, and their public health service capabilities are stronger. From a geographical perspective, Western Europe and Northern Europe, where the overall economic development level is relatively high, have relatively strong public health service capabilities. Therefore, the level of economic development is the top priority for improving the public health service capacity. It is necessary to promote economic system reform through the development of science and technology and strengthening the infrastructure construction. Promoting the development of the public health service system through continuous economic development is an essential choice.

The level of urbanization is negatively correlated with the capacity of public health services, which is consistent with the phenomenon of “reverse urbanization” that has generally appeared in Europe. People with a certain economic level have a preference for the rural environment. Urban people buy land and houses in rural areas one after another, causing the population to flow back from urban to rural areas, resulting in a decline in the urbanization rate [39]. Countries with high levels of economic development and public health service capabilities, such as Germany, France and Italy, all have a low urbanization rate. In addition, countries with high urbanization rates have large urban populations and relatively dense distribution. This puts greater pressure on regional public health services, and, to a certain extent hinders, the improvement of public health service capabilities. Therefore, we should rationally plan the distribution of urban population, control the urbanization rate in relevant areas of the country, and ensure the effective supply and steady development of public health services [40]. 

Studies have shown that technological innovation can obviously promote the improvement of public health service capacity. Technology is an important driving force for the progress and development of human society, bringing advanced medical equipment, diagnostic technology and medical resources which are more tailored to the needs of the country [41]. This can meet the individual needs of people regarding public health services. Countries in the EU with stronger technology, such as Germany, Austria and Sweden, have strong public health service capabilities. Therefore, to improve the public health service capacity, it is necessary to continuously carry out technological innovation with service as the center and increase the investment in research and development funds. We ought to introduce, produce and use advanced materials to meet individual needs for public health services [42].

There is a positive correlation between the degree of aging and the capacity of public health services. This indicates that countries of the EU with a higher degree of aging now have relatively strong public health service capacities, which are in line with the reality of the EU. The degree of aging is closely related to the capacity of public health services. In the EU, Portugal, Spain, Germany, Greece and Finland are countries with more serious aging. The public health service capacity of these countries is relatively strong. This is because the more serious the aging, the greater the demand for public health services in the country. As a result, the government will invest in a series of elements such as funds, manpower and materials to improve public health service capabilities. Therefore, to a certain extent, public health service capacity is positively correlated with the degree of aging. However, it is still important to reasonably control the number of aging population to ensure sufficient labor force in the country.

## 5. Analysis of COVID-19 Epidemic and Public Health Service Capacity

**Figure 2:**
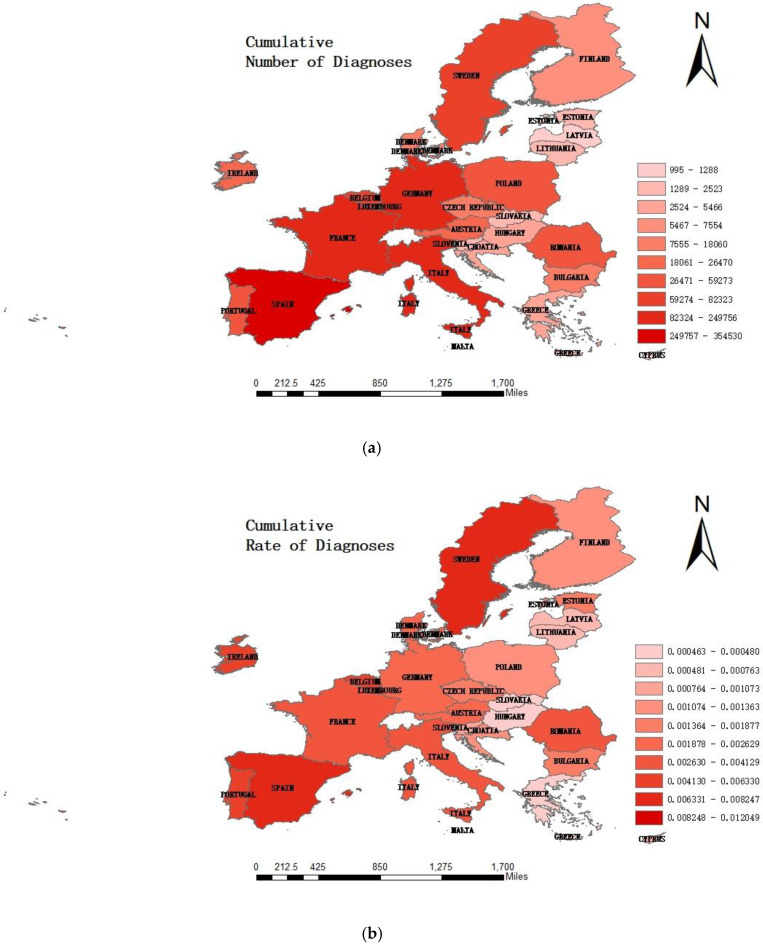

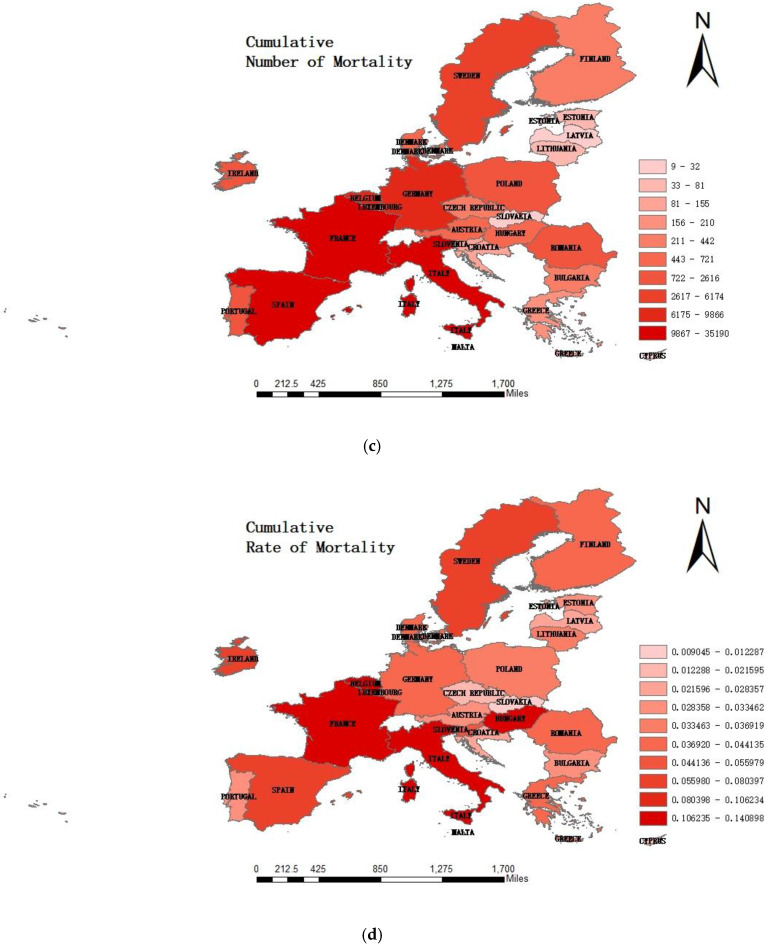
The graduated color figures of the EU about cumulative number of diagnoses (**a**), cumulative rate of diagnoses (**b**), cumulative number of mortality (**c**) and cumulative rate of mortality (**d**) (by 8 August 2020).

Europe is one of the hardest hit areas of the COVID-19 epidemic that has swept the world. Countries in the EU account for the majority of the countries with the most confirmed cases. None of the 27 member states have been spared. According to the data from official announcements of the World Health Organization and governments, the epidemics in Italy, Spain, Germany and France are very serious. As can be seen from Figure 2 below, among the EU, countries in Western European with more developed economies and strong public health service capabilities are the hardest hit areas, such as Italy, Spain, Germany and France. The cumulative number of confirmed cases has reached more than 200,000. Spain is the most serious, with a cumulative number of diagnoses of 354,530. Countries in Eastern European with weaker public health service capabilities are generally lighter, such as Cyprus and Latvia, where the cumulative number of confirmed cases is less than 1300. The epidemic reflects the public health service capacity indirectly. However, this is also closely connected to the emergency response mechanism and the government’s ability to judge, make decisions and implement public policies [43].

A correlation analysis between the public health service capacity of the EU and the cumulative number of diagnoses, cumulative rate of diagnoses, cumulative numbers of cures, cumulative number of mortality and cumulative rate of mortality under the epidemic of COVID-19, Table 5, was obtained. The public health service capacity of the EU is positively correlated with them. The correlation with the cumulative number of diagnoses is stronger. According to habitual thinking and the preliminary results of this study, countries with higher public health service capabilities have relatively more developed economies. The situation of a country’s epidemic should be more optimistic. The results violated common sense. It can be inferred that the countries with severe epidemics are countries with a strong public health service capacity [44,45]. Considering the existing research, this paper believes that the possible reasons are as follows.

First, the detection capabilities of countries. On 16 March 2020, the director of the World Health Organization issued a call for enhanced testing in Geneva. Detection plays an important role in epidemic control and transmission. Germany’s public health service capacity is very strong. The diagnosis rate of COVID-19 in Germany is 2.6‰, and the mortality rate is 4.26%. Lothar Wieler, director of the Robert Koch Institute in Germany, believes that the low mortality rate is due to the high level of detection capabilities in Germany. He revealed that German laboratories conduct 1.6 million virus detection for COVID-19 every week. Since the outbreak, the total number of detection in some European countries is probably less than that in Germany in a week. The high-level detection can identify individuals with few symptoms, making it possible for Germany to have fewer undetected infections than in other countries. In the early stages of the epidemic, large-scale detection can indeed reduce mortality. Because public health institutions can find more cases of infection and treat them as soon as possible, the chances of survival are much higher. In addition, the detection system does not make it easy to let go of the patients with mild or no symptoms, and immediately block the social contact of these patients. Compared with those countries with lower economies, countries with stronger economic power have stronger public health service capabilities, correspondingly stronger detection capabilities, and more resources and capabilities to detect infected patients. This has a direct and positive effect on the number of diagnoses [46].

Second is the high probability of spreading COVID-19 epidemic. The first country in Europe to have severe outbreaks were Italy and Spain [47]. European countries such as Italy and Spain, which are tourist destinations, have a temperate maritime climate, with no severe cold in winter and no scorching heat in summer, and the climate is suitable. A large number of scenic spots attract tourists from all over the world. The outbreak of the epidemic coincided with winter, which was a good time to travel and accelerated the spread of the epidemic. In addition, the population distribution of these countries is relatively concentrated and dense, making it easier to spread the epidemic.

Third, the integrated system of the EU. This epidemic is not only a great challenge to the public health security of European, but also a severe test of European integration. Europe is one of the hardest hit areas of the COVID-19 epidemic that has swept the world. No member of the EU was spared, with Italy and Spain experiencing the worst outbreaks. Countries of the EU do not need to apply for complicated procedures such as visas, and can easily realize the cross-border population flow. Compared with other independent countries, their large-scale and wide-scale population movements aggravated the spread of the epidemic to a certain extent [48].

Fourth, the inefficient implementation of government policy. First of all, the number of deaths in Italy, Spain, France, etc., is around 30,000. These countries have adopted extensive and strict restrictive measures, such as suspension of work and business. However, the relevant policies were not promulgated in a timely manner and failed to contain the spread of the epidemic at the best time, which led to the increase in the number of diagnoses and mortality. Secondly, Sweden, Denmark, Greece, Portugal and Netherlands and other countries did not impose comprehensive restrictions. However, most of the countries have fewer than 1000 deaths. Moreover, many countries underestimated the severity of the epidemic. Compared with other European powers, Germany is more serious, rigorous and conscious, and has a higher sense of crisis. While other countries have not taken action or banned the gathering of more than 10 people, Germany has already issued a ban on more than two people. In addition, when the epidemic was serious, Italy, Spain, Britain, France and other countries reported that people still hold assembly, barbecues and dinners in spite of the ban, but few German people have been reported to do so. It can be seen that these countries with a more serious epidemic situation in the EU underestimated the severity of the epidemic, resulting in the formulation of restriction decrees not occurring in a timely manner [49]. This is due to the concept of democracy and freedom in European people’s nature. They are not accustomed to the relevant restrictive decrees, which have not been effectively implemented. People are still engaged in gathering activities which accelerate the spread of the epidemic.

Fifth, the adverse selection of youth. The outbreak began in Italy in mid-February. At that time, the epidemic of Italian was mainly concentrated in the northern Lombardy and Veneto regions. In order to prevent the spread of the epidemic of COVID-19, Italy adopted quarantine measures for 50,000 people, and even began to adopt restrictions on “closing cities and roads”. Late February is a time for carnivals all over Europe. In order to celebrate the “holiday”, people gather to play and have fun more frequently. Most young people’s mentality upholds the bad mentality of early illness, early treatment and early end. They failed to implement effective epidemic prevention and control, resulting in a violent increase in patients in the later period. This provides a reference for other countries in European. Governments have successively promulgated various policies to promote the dangers of COVID-19 and the importance of prevention and control [50].

## 6. Conclusions

The global epidemic is still worsening, and the EU has a global leadership in defining the “new normal” as part of the global recovery. In the context of the global epidemic of COVID-19, it is particularly important to evaluate the EU’s public health service capacity and explore its driving factors. The paper selected data from three dimensions of public health expenditure, medical care resources, and government emergency coordination capabilities to measure the public health service capabilities of the EU from the period 2008 to 2017. The overall public health service capacity has shown a gradual growth trend, which shows that the EU paid more and more attention to public health services in recent years. Countries with strong public health capabilities are Germany, France, Italy, the Netherlands and Spain. The countries with weaker capabilities than that are Cyprus, Estonia, Latvia and Bulgaria. The capacity of public health services in Western Europe is higher than that of Northern Europe, Southern Europe and Eastern Europe. Although the overall public health service capacity of Western Europe is relatively high, the development is not balanced and stable. The paper used the Tobit panel model to explore the driving factors of public health service capacity. Specifically, population density is positively related to this. Countries with higher population density can not only trigger the agglomeration effect of funds, goods and talents, but also have stronger radiation capacity in the hinterland. Opening up promotes the improvement in public health service capabilities by affecting the optimal allocation of production factors and resources. Because of the demand for high-level public health services from the talents, the level of education can effectively promote its development. Only with economic strength can a more complete public health service system be established. The level of urbanization is negatively correlated with this, which is consistent with the phenomenon of “reverse urbanization” that has generally appeared in Europe. Technology brings more medical resources that can meet more individual needs. The degree of aging is positively related. The more serious the degree of aging, the greater the demand for public health services the country has to deal with. The government will adopt a series of investment of funds, manpower, materials and other elements to improve public health service capabilities.

Through the comparative analysis of the COVID-19 epidemic and public health service capacity, it can be concluded that the epidemic situation in countries with a strong public health service capacity is more severe. The possible reasons are as follows. First, the detection capabilities of countries: countries with stronger economic power and public health service capacities have more resources and a greater capacity to detect infected patients. Second, the high probability of spreading the COVID-19 epidemic: countries with a severe epidemic, such as Italy and Spain, have a favorable climate and developed tourism. Their population distribution is very dense, and it is easy to spread the epidemic. Third, the integrated system of the EU: countries in the EU can easily realize cross-border population mobility without going through complicated procedures. The large-scale and wide-ranging population movement has intensified the spread of the epidemic to a certain extent. Fourth, the inefficient implementation of government policy: many countries underestimated the severity of the COVID-19 epidemic. The restriction decrees formulated by governments were not timely enough, and the concept of democracy and freedom in the nature of European people led to the ineffective implementation of these decrees and accelerated the spread of the epidemic. Fifth, the adverse selection of youth: most of the young hold the bad mentality of early illness, early treatment and early end. They have not carried out effective epidemic prevention and control, resulting in a sharp increase in patients in the later period.

In view of the above conclusions, we should improve the public health service capacity of the EU from the following aspects. First, while increasing the popularity of public health services, improve the supply mechanism of public health resources, which will increase the utilization rate of medical resources and promote the long-term development of national public health services. Second, we should improve the degree of opening up, promote economic exchanges and cooperation, attract foreign investment, and introduce medical personnel, advanced technology and management experience. Third, it is necessary to increase the investment related to public health services, carry out service-oriented technological innovation, and constantly improve the national virus detection capacity and the government’s emergency response capability. Fourth, we ought to constantly strengthen talent education, pay more attention to the cultivation of high-quality talents, promote the exchange and cooperation of talents, and meet the personalized needs of public health services. Fifth, ensure that the country’s labor force, reasonable planning of urban population distribution and reasonable control of the proportion of aging are sufficient. There are still many areas to be explored and discussed in the future research. The public health services of the EU under COVID-19 have failed to play their due role. The governments of the EU have not properly mobilized public health service resources, and the public health service system needs to be further improved. This is closely related to the government system and the promulgation of relevant laws and regulations. Countries in the EU should pay attention to this COVID-19 epidemic and take response measures as soon as possible. The elderly with weak resistance are the main groups of the epidemic. However, the relationship between the death of the elderly population and the capacity of public health services was not considered in the comparative analysis. Future research should take the number of elderly deaths under COVID-19 as an indicator, and explore the impact of governance capacity and laws and regulations on the capacity of public health services.

## Figures and Tables

**Table 1:** Public health service capacity evaluation index system.

First Level Index	Second Level Index	Third Level Index
Public Health Service Capacity	Public Health Expenditure	Current Health Expenditure as Percentage of GDP $X_{11}$
		Percentage of Domestic Government Health Expenditure in GDP $X_{12}$
		Current Health Expenditure Per Capita $X_{13}$
		Percentage of Government Health Expenditure in Total Government Expenditure $X_{14}$
		Proportion of Health Care in Residents’ Consumption Expenditure $X_{15}$
	Medical Care Resources	Number of Doctors Per 10,000 People $X_{21}$
		Number of Specialists $X_{22}$
		Number of Nursing Staff $X_{23}$
		Number of Published Medical Papers $X_{24}$
		Number of Beds Per 1000 People $X_{25}$
	Government Emergency Coordination Capabilities	Score of Legislation $X_{31}$
		Score of Coordination $X_{32}$
		Score of Surveillance $X_{33}$
		Score of Response $X_{34}$
		Score of Preparedness $X_{35}$

Note: Data from “International Statistical Yearbook” and World Health Organization.

**Table 2:** EU public health service capacity scores based on principal component analysis.

Province	2008	2009	2010	2011	2012	2013	2014	2015	2016	2017
Austria	0.756	0.755	0.746	0.737	0.759	0.756	0.773	0.784	0.779	0.727
Belgium	0.718	0.724	0.722	0.671	0.711	0.702	0.716	0.714	0.715	0.720
Bulgaria	0.542	0.546	0.570	0.583	0.602	0.618	0.650	0.662	0.678	0.679
Cyprus	0.518	0.516	0.510	0.507	0.549	0.595	0.593	0.629	0.672	0.667
Croatia	0.710	0.722	0.732	0.719	0.725	0.716	0.636	0.636	0.635	0.633
Czechia	0.697	0.715	0.716	0.723	0.733	0.731	0.731	0.727	0.738	0.750
Denmark	0.699	0.708	0.703	0.683	0.716	0.766	0.774	0.774	0.770	0.753
Estonia	0.637	0.624	0.606	0.588	0.580	0.607	0.621	0.626	0.628	0.618
Finland	0.678	0.690	0.700	0.726	0.751	0.711	0.719	0.751	0.756	0.757
France	0.727	0.739	0.758	0.821	0.822	0.842	0.846	0.853	0.862	0.862
Germany	0.860	0.882	0.889	0.923	0.926	0.941	0.955	0.960	0.967	0.972
Greece	0.666	0.679	0.679	0.680	0.659	0.659	0.663	0.650	0.654	0.653
Hungary	0.582	0.602	0.632	0.691	0.643	0.718	0.706	0.718	0.717	0.716
Ireland	0.649	0.647	0.628	0.622	0.698	0.694	0.683	0.697	0.699	0.700
Italy	0.693	0.696	0.692	0.728	0.747	0.733	0.736	0.758	0.790	0.792
Latvia	0.488	0.530	0.573	0.622	0.660	0.659	0.659	0.655	0.669	0.677
Lithuania	0.611	0.624	0.634	0.653	0.660	0.670	0.679	0.693	0.689	0.693
Luxembourg	0.526	0.538	0.565	0.623	0.595	0.696	0.695	0.692	0.691	0.692
Malta	0.543	0.568	0.589	0.624	0.693	0.704	0.721	0.716	0.714	0.717
The Netherlands	0.773	0.779	0.783	0.788	0.795	0.808	0.815	0.820	0.823	0.824
Poland	0.583	0.607	0.633	0.689	0.700	0.715	0.700	0.702	0.706	0.709
Portugal	0.762	0.766	0.766	0.762	0.757	0.756	0.754	0.753	0.741	0.741
Romania	0.597	0.604	0.637	0.648	0.639	0.635	0.662	0.666	0.666	0.678
Slovakia	0.541	0.593	0.661	0.699	0.731	0.745	0.741	0.738	0.741	0.737
Slovenia	0.630	0.627	0.635	0.638	0.628	0.629	0.659	0.692	0.700	0.719
Spain	0.604	0.627	0.621	0.647	0.774	0.771	0.790	0.787	0.798	0.799
Sweden	0.685	0.708	0.722	0.780	0.804	0.778	0.780	0.778	0.777	0.776

**Table 3:** Regression Analysis Index of European Union (EU) Public Health Service Capacity.

Dimension	Variable
Population Density	Population Density $y_{1}$
Degree of Opening Up	Globalization Index $y_{2}$
Education Level	Average Years of Education Per Capita $y_{3}$
Economic Development Level	GDP and Per Capita GDP $y_{4}$
Urbanization Level	Urbanization Rate $y_{5}$
Technological Innovation Level	R&D Investment as Percentage of GDP $y_{6}$
Gap between the Rich and the Poor	Gini Coefficient $y_{7}$
Degree of Aging	Proportion of People over 65 Years Old $y_{8}$

Note: Data from “International Statistical Yearbook” and World Health Organization.

**Table 4:** Regression results of driving factors of EU public health service capacity.

Variable	Estimated Coefficient	*Z* Value	Significance	Estimated Coefficient	*Z* Value	Significance
	Tobit (Random Effects)			Tobit (Fixed Effects)		
Population Density	0.045	1.73	*	0.040	4.60	***
Globalization Index	0.008	0.55		0.050	6.10	***
Average Years of Education Per Capita	0.009	1.36		0.020	2.66	***
GDP	0.048	2.14	**	0.074	8.04	***
Urbanization Rate	−0.021	−0.79		−0.036	−3.90	***
Per Capita GDP	0.023	1.19		0.002	0.39	
R&D Investment as Percentage of GDP	0.034	1.78	*	0.044	3.92	***
Gini Coefficient	0.026	1.74	*	0.005	0.56	
Proportion of People over 65 Years Old	0.111	8.30	***	0.057	6.17	***
_cons	0.437	19.57	***			
sigma_u	0.114	4.74	***			
sigma_e	0.074	20.7	***			

Note: *Z* Value (*** *p* < 0.01, ** *p* < 0.05, * *p* < 0.1).

**Table 5:** Correlation of the COVID-19 Epidemic and Public Health Service Capacity.

	Cumulative Number of Diagnoses	Cumulative Rate of Diagnoses	Cumulative Number of Mortality	Cumulative Rate of Mortality	Cumulative Number of Cures	Public Health Service Capacity Scores
Cumulative Number of Diagnoses	1	0.38	0.33	0.22	0.26	0.27
Cumulative Rate of Diagnoses	0.38	1	0.92	0.54	0.92	0.69
Cumulative Number of Mortality	0.33	0.92	1	0.69	0.81	0.57
Cumulative Rate of Mortality	0.22	0.54	0.69	1	0.39	0.43
Cumulative Number of Cures	0.26	0.92	0.81	0.39	1	0.66
Public Health Service Capacity Scores	0.27	0.69	0.57	0.43	0.66	1

## Appendix A

**Table A1:** EU public health service capacity scores based on factor analysis.

Province	2008	2009	2010	2011	2012	2013	2014	2015	2016	2017
Austria	0.665	0.668	0.662	0.659	0.677	0.678	0.691	0.697	0.694	0.656
Belgium	0.636	0.648	0.644	0.611	0.641	0.638	0.650	0.645	0.647	0.653
Bulgaria	0.427	0.430	0.457	0.467	0.486	0.498	0.530	0.536	0.548	0.549
Cyprus	0.390	0.394	0.391	0.392	0.423	0.459	0.456	0.480	0.511	0.504
Croatia	0.590	0.603	0.610	0.591	0.598	0.580	0.520	0.520	0.520	0.518
Czechia	0.570	0.595	0.590	0.598	0.604	0.612	0.612	0.604	0.612	0.622
Denmark	0.626	0.645	0.635	0.621	0.645	0.687	0.695	0.691	0.689	0.677
Estonia	0.507	0.502	0.485	0.468	0.463	0.486	0.498	0.504	0.507	0.499
Finland	0.581	0.596	0.603	0.625	0.647	0.611	0.627	0.650	0.652	0.654
France	0.689	0.707	0.720	0.768	0.770	0.790	0.797	0.800	0.809	0.810
Germany	0.827	0.857	0.862	0.889	0.894	0.912	0.928	0.934	0.944	0.953
Greece	0.572	0.591	0.588	0.584	0.559	0.548	0.551	0.540	0.547	0.545
Hungary	0.471	0.488	0.516	0.560	0.524	0.578	0.568	0.577	0.578	0.577
Ireland	0.579	0.589	0.562	0.565	0.621	0.614	0.597	0.584	0.588	0.589
Italy	0.633	0.640	0.638	0.661	0.674	0.666	0.671	0.686	0.714	0.716
Latvia	0.382	0.411	0.441	0.475	0.505	0.504	0.505	0.504	0.518	0.522
Lithuania	0.493	0.511	0.511	0.526	0.527	0.535	0.546	0.561	0.564	0.568
Luxembourg	0.465	0.479	0.494	0.524	0.506	0.573	0.573	0.566	0.565	0.566
Malta	0.453	0.468	0.484	0.523	0.577	0.589	0.606	0.601	0.601	0.607
The Netherlands	0.682	0.693	0.701	0.709	0.720	0.734	0.741	0.739	0.742	0.742
Poland	0.482	0.502	0.518	0.557	0.566	0.580	0.570	0.572	0.578	0.583
Portugal	0.654	0.662	0.660	0.653	0.644	0.641	0.638	0.634	0.626	0.626
Romania	0.471	0.478	0.510	0.505	0.502	0.508	0.527	0.528	0.528	0.542
Slovakia	0.457	0.503	0.553	0.577	0.604	0.614	0.606	0.601	0.607	0.601
Slovenia	0.524	0.530	0.536	0.539	0.533	0.527	0.552	0.580	0.592	0.608
Spain	0.545	0.572	0.568	0.588	0.682	0.682	0.696	0.695	0.705	0.707
Sweden	0.601	0.623	0.630	0.715	0.735	0.720	0.723	0.717	0.716	0.716
